# A new pan-chelydrid turtle, *Tavachelydra stevensoni* gen. et sp. nov., from the lower Paleocene (early Danian, Puercan) Corral Bluffs Study Area in the Denver Basin, Colorado

**DOI:** 10.1186/s13358-025-00375-4

**Published:** 2025-08-05

**Authors:** Tyler R. Lyson, Holger Petermann, Salvador Bastien, Natalie Toth, Evan Tamez-Galvan, Sadie M. Sherman, Walter G. Joyce

**Affiliations:** 1 Department of Earth Sciences, Denver Museum of Nature & Science, Denver, Colorado 80205 USA; 2 Department of Geosciences, University of Fribourg, 1700 Fribourg, Switzerland

**Keywords:** Testudines, Chelydroidea, Pan-Chelydridae, Taphonomy, Durophagy

## Abstract

Isolated pan-chelydrid turtle shell fragments are common in Late Cretaceous and early Paleocene sediments across western North America, but more complete and associated specimens are rare, obfuscating our understanding of the group’s early evolution. Here we describe a new genus and species, *Tavachelydra stevensoni*, of stem-chelydrid turtle from the early Paleocene of the Denver Formation (Danian, Puercan I and II) of Colorado based on complete shells, associated pelvic material, and referred cranial material. Our phylogenetic analysis places *T. stevensoni* as the immediate sister to crown chelydrids based on, among others, a purely ligamentous attachment of the plastron and carapace. The costiform process of the nuchal, an important character complex in chelydroid turtles, shows variation in either ending in peripheral II or III. The *T. stevensoni* material was mostly found in laminated fine-grained deposits, suggesting this taxon inhabited ponded-water environments. The referred cranial material shows broad triturating surfaces indicating a durophagous diet, further underscoring durophagy as an important feeding strategy during the early Paleocene.

**Supplementary Information:**

The online version contains supplementary material available at 10.1186/s13358-025-00375-4.

## Introduction

The clade of extant chelydrid turtles (*Chelydridae*) includes five species that are endemic to the New World (TTWG, 2021) and whose combined geographic ranges extend from northern South America to southern Canada. While not a diverse clade, chelydrid turtles are a common component of most North American freshwater ecosystems. The total group of chelydrids (i.e., *Pan-Chelydridae* sensu Joyce et al., 2021) is found throughout Laurasia, but fossil remains are notoriously fragmentary, likely due to the thin nature of the carapace and the fact that pan-chelydrid shells are not tightly sutured together and commonly fall apart soon after death (Hutchison, 2008; Joyce, 2016; Joyce et al., 2022a). Fragments of pan-chelydrids have been reported from several Late Cretaceous (Turonian through Maastrichtian) formations throughout the Rocky Mountain and Great Plains regions, but because of their fragmentary nature, they have not been described or figured in any detail (Hutchison, 2008). The earliest described and figured pan-chelydrid turtles are *Denverus middletoni* Hutchison & Holroyd, 2003 and *Protochelydra zangerli* Erickson, 1973. *Denverus middletoni* is based on a fragmentary shell from the early Paleocene (Puercan North American Land Mammal Age [NALMA]) Corral Bluffs Study Area in the Denver Basin (Hutchison & Holroyd, 2003) while *P. zangerli* is based on abundant, though poorly described shell, cranial, and postcranial material from the early Paleocene, Tiffanian NALMA, Wannagan Creek quarry in the Williston Basin (Erickson, 1973, 2010). Well-preserved pan-chelydrid material from the early Paleocene Puercan and Torrejonian NALMAs are otherwise lacking.

**Fig. 1:**
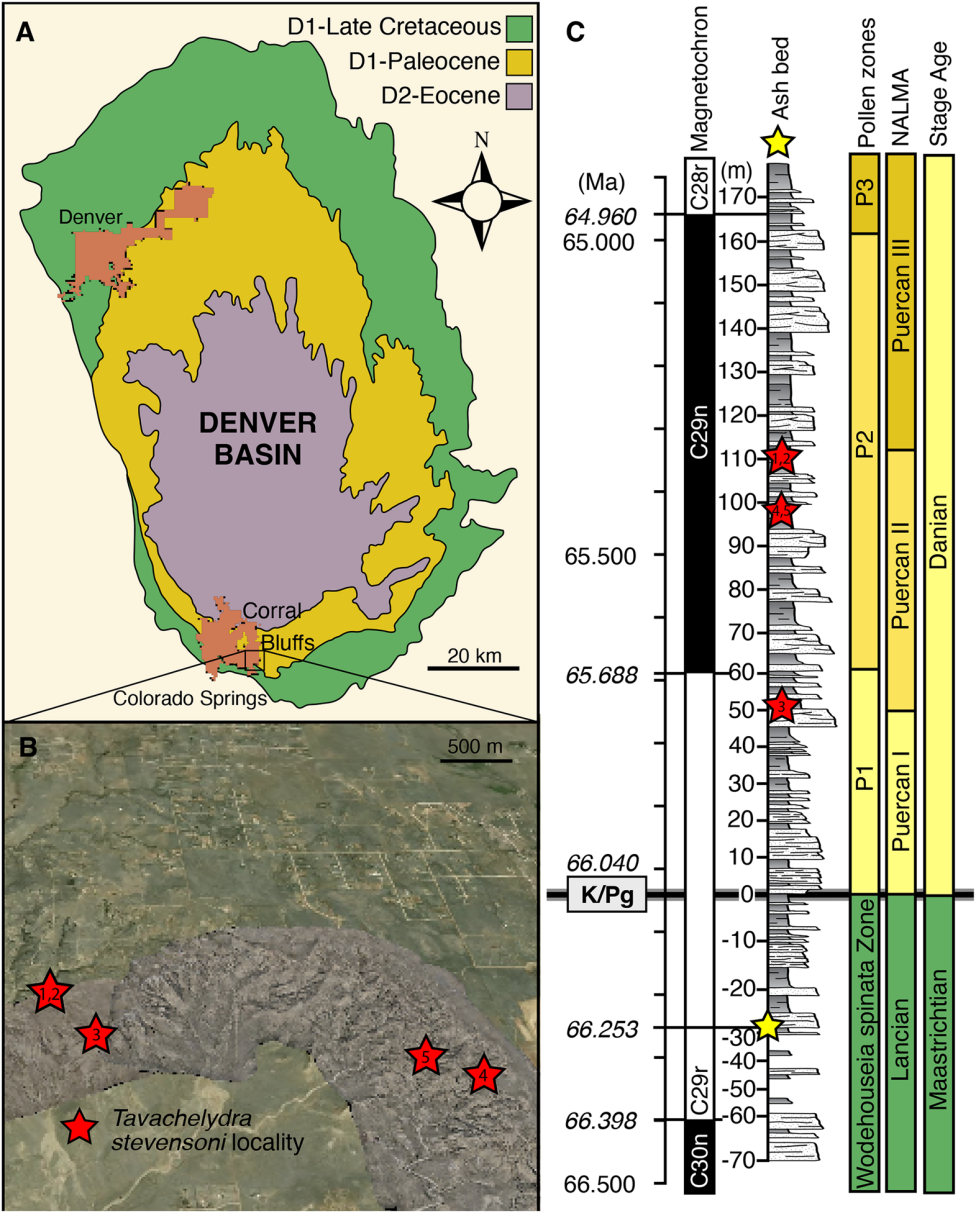
Geography, chronostratigraphy, and biostratigraphy of the Corral Bluffs Study Area within the Denver Basin from which specimens of *Tavachelydra stevensoni* were collected. **A**, map of Late Cretaceous through Eocene sediments within the Denver Basin showing the location of the Corral Bluffs Study Area within Colorado Springs (highlighted by box and enlarged in part **B**) in the southwestern portion of the basin. **B**, high-resolution photogrammetric model of the eastern portion of the Corral Bluffs Study Area overlain on Google Earth with geographic locations of *T. stevensoni* denoted by red stars: **1**, DMNH EPV.141854/DMNH Loc.19258; **2**, DMNH EPV.143100/DMNH Loc. 20,053; **3**, DMNH. EPV.134087/DMNH Loc. 7082; **4**, DMNH. EPV.136265/DMNH Loc. 18,852; **5**, DMNH EPV.143200/DMNH Loc. 6284. **C**, age, magnetostratigraphic, lithostratigraphic, and biostratigraphic logs showing the stratigraphic placement of *T. stevensoni* localities (red stars; see numbers from **B**). Stratigraphy is tied to the Geomagnetic Polarity Time Scale (GPTS2012; Gradstein et al., 2012; Ogg, 2012) using remnant magnetization of the rocks at the Corral Bluffs Study Area, two CA-ID-TIMS U–Pb-dated volcanic ash beds (yellow star; two dated ash samples represent the same volcanic ash locality and thus only one yellow star), and the palynologically defined K/Pg boundary (italicized dates) (Fuentes et al., 2019; Lyson et al., 2019). The lithostratigraphic log is a composite and shows that the sequence is dominated by intercalated mudstone and sandstone, reflecting a loosely anastomosing fluvial environment (Lyson et al., 2019). Pollen biozones follow Nichols and Fleming (2002) and are defined by diversification of *Momipites* spp. (fossil juglandaceous pollen). North American Land Mammal ‘ages’ (NALMA) follows Lyson et al. (2019) as defined by Lofgren et al. (2004). *Ma*, million years ago; *K/Pg*, Cretaceous-Paleogene boundary

Recent collecting efforts in the Corral Bluffs Study Area in the Denver Basin of North America (Fig. 1) have yielded a wealth of remarkably well-preserved vertebrate fossils from the early Paleocene Puercan NALMA, including mammals (Krause et al., 2021; Bertrand, 2022; Weaver et al., 2024), crocodilians (Lessner et al., 2025; Lyson et al., 2019), and turtles (Lyson et al., 2021a, 2021b; Petermann et al., 2022). Many of the fossils are preserved in phosphatic concretions, a mode of preservation that is unusual in terrestrial environments (Lyson et al., 2019). Here, we provide a detailed description of a new species of pan-chelydrid from the Corral Bluffs Study Area based on a well-preserved shell and associated pelvis, a well-preserved carapace, and two poorly preserved crania.

## Methods

### Manual preparation

Pin vises, air scribes, and air abrasion with sodium bicarbonate and dolomite abrasive were used to remove matrix from the fossils. The material was consolidated with Paraloid B72 and cyanoacrylates. Preparation of the *Tavachelydra stevensoni* material was completed by S. Sherman, S. Bastien, N. Brandborg, and N. Toth at the Denver Museum of Nature & Science, as well as contract preparators R. Masek and A. Lujan.

### Surface scanning

The anterior region of the carapace of DMNH EPV.141854 and the entire carapace of DMNH EPV.143100 were surface scanned using the Artec Space Spider and Artec Leo, respectively. 3D surface models were generated in the software Artec Studio 17 Professional and both models are available for download at MorphoSource (project ID: 000730318). Surface scanning and digital processing was completed by E. Tamez-Galvan.

## Geological settings and age control

All described material is from the early Paleocene D1 sequence in the Denver Basin, Colorado (Fig. 1). Each specimen is from the Corral Bluffs Study Area, which is located in El Paso County in south-central Colorado, east of Colorado Springs. The Corral Bluffs Study Area is in the southern portion of the Denver Basin, which contains sediments from the Upper Cretaceous to the Eocene (Raynolds & Johnson, 2003). The Corral Bluffs study area is a south-facing arc, or “corral”, of exposed rock that contains the best outcrop of the D1 sequence of the Denver Formation (Raynolds, 1997, 2002), which is latest Cretaceous to earliest Paleocene in age. Recent chronostratigraphic work at Corral Bluffs found magnetochrons 30n through 28r, with most of the cliff-forming and fossil-bearing outcrop occurring within magnetochron 29n (Fuentes et al., 2019; Hicks et al., 2003). In addition to the magnetochrons, a pollen-defined Cretaceous-Paleogene (K/Pg) boundary has been identified in two stratigraphic sections that span the entire distance of the outcrop at Corral Bluffs. Finally, two independent, but nearly identical ^206^Pb/^238^U age estimates from the same volcanic ash provide an additional calibration point (Fuentes et al., 2019). The radiometric date, K/Pg boundary, and three magnetic reversals (30n/29r, 29r/29n, and 29n/28r), along with the overall stratigraphic thickness between each of these dated time horizons provide the basis for the model used to determine the age of fossils found at the Corral Bluffs Study Area (Fuentes et al., 2019; Lyson et al., 2019; Fig. 1). For the magnetic reversals and K/Pg boundary, we use the 2012 Geomagnetic Polarity Time Scale (GPTS; Gradstein et al., 2012; Ogg, 2012), as well as a second age model developed from the eastern portion of the Denver Basin, which has abundant ashes tightly bracketing the K/Pg and relevant magnetochrons (Clyde et al., 2016). Age estimates for each time horizon in the GPTS 2012 age model (Gradstein et al., 2012; Ogg, 2012) are as follows: C29n/C28r at 64.958 Ma, C29r/C29n at 65.688 Ma, the K/Pg boundary at 66.04 Ma, and C30n/C29r at 66.398 Ma. For Clyde et al. (2016) ages are C29n/C28r at 64.893 Ma ± 0.056 my, C29r/C29n at 65.806 Ma ± 0.048 my, the K/Pg boundary at 66.021 Ma ± 0.024 my, and C30n/C29r at 66.436 Ma ± 0.039 my.

Recent biostratigraphic work (Fuentes et al., 2019; Lyson et al., 2019) at the Corral Bluffs Study Area identified the index mammal taxa for the Puercan II and Puercan III NALMAs (Lofgren et al., 2004) and the earliest Paleocene P1 through P3 pollen biozones (Lyson et al., 2019). All specimens described herein are stratigraphically within the Puercan I and II NALMAs and the P1 and P2 pollen biozones.

## Systematic paleontology

*Testudines* Batsch, 1788 (sensu Joyce et al., 2020a).

*Cryptodira* Cope, 1868 (sensu Joyce et al., 2020b).

*Chelydroidea* Baur, 1893 (sensu Joyce et al., 2021).

*Pan-Chelydridae* Joyce et al., 2004 (sensu Joyce et al., 2021).

**Fig. 2:**
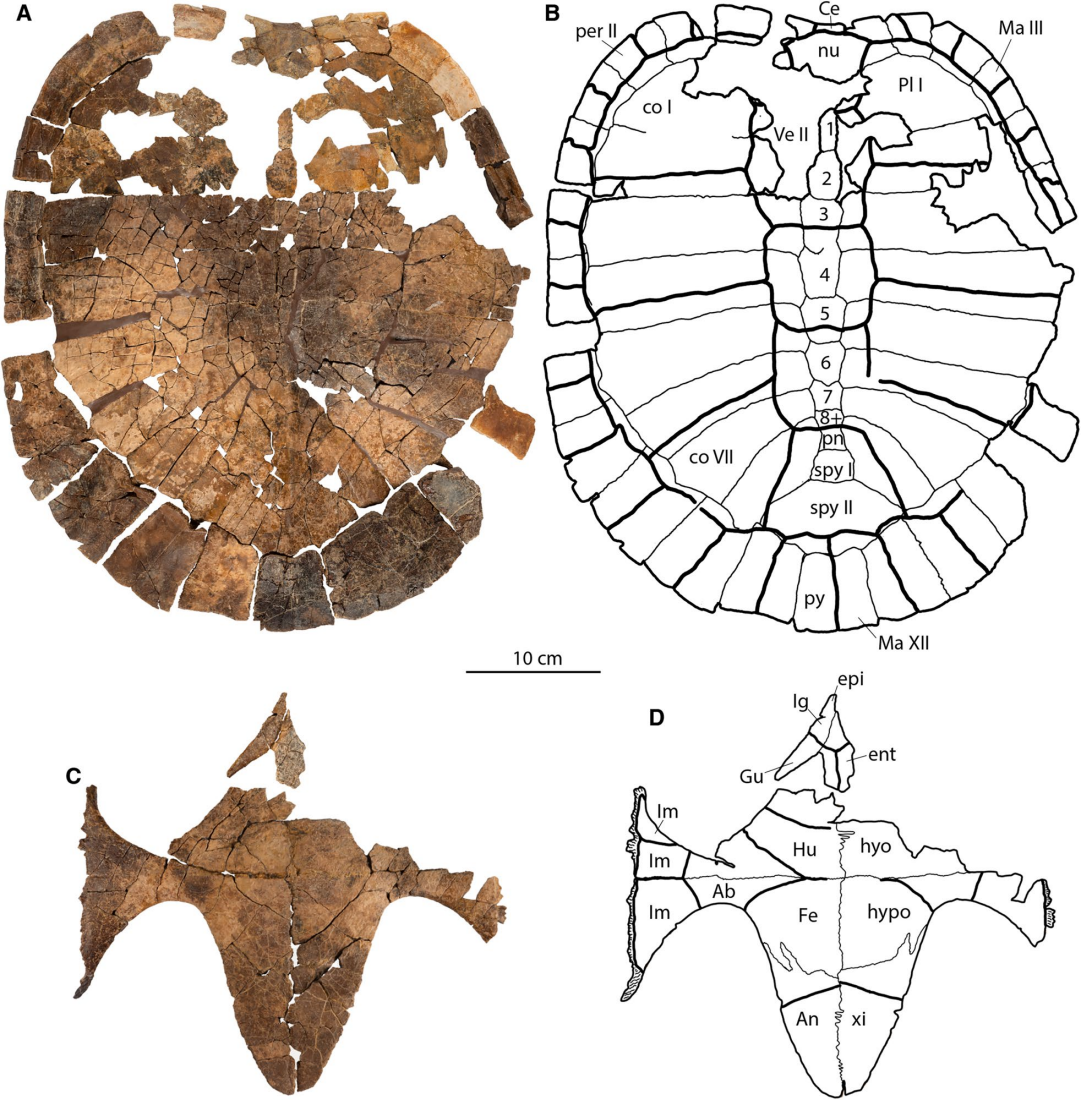
*Tavachelydra stevensoni*, gen. et sp. nov., DMNH EPV.141854 (DMNH Loc.19258), holotype, external view of shell. **A**, photograph and **B**, interpretive line drawing of the carapace. **C**, photograph and **D**, interpretive line drawing of the plastron. *Ab* abdominal scale, *An* anal scale, *Ce* cervical scale, *co* costal, *ent* entoplastron, *epi* epiplastron, *Fe* femoral scale, *Gu* gular scale, *Hu* humeral scale, *hyo* hyoplastron, *hypo* hypoplastron, *Ig* intergular scale, *Im* inframarginal scale, *Ma* marginal scale, *nu* nuchal, *per* peripheral, *Pl* pleural scale, *pn* postneural, *spy* suprapygal, *py* pygal, *Ve* vertebral scale, *xi* xiphiplastron. Arabic numerals denote neurals

**Fig. 3:**
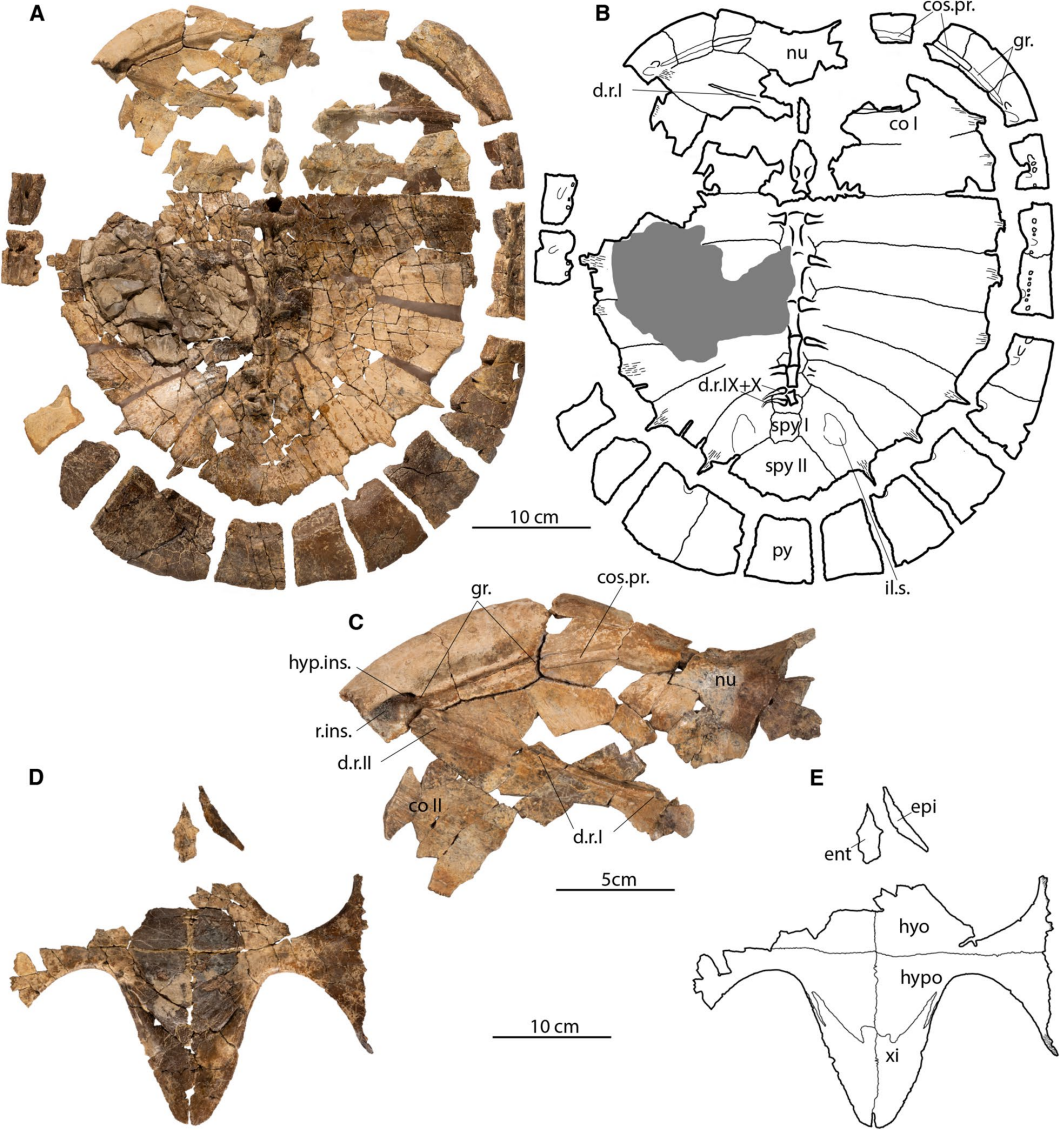
*Tavachelydra stevensoni*, gen. et sp. nov., DMNH EPV.141854 (DMNH Loc.19258), holotype, internal view of shell.** A**, photograph and **B**, interpretive line drawing of the carapace. **C**, closeup photograph of the anterolateral region of the carapace highlighting the costiform process of nuchal superficially extending across peripherals I and II and ending in peripheral III. **D**, photograph and **E**, interpretive line drawing of the plastron. *co* costal, *cos.pr.* costiform process, *d.r.* dorsal rib, *ent* entoplastron, *epi* epiplastron, *gr.* groove, *hyo* hyoplastron, *hyo.ins.* hyoplastral insertion, *hypo* hypoplastron, *il.s.* iliac scar, *nu* nuchal, *py* pygal, *r.ins.* rib insertion, *spy* suprapygal, *xi* xiphiplastron

**Fig. 4:**
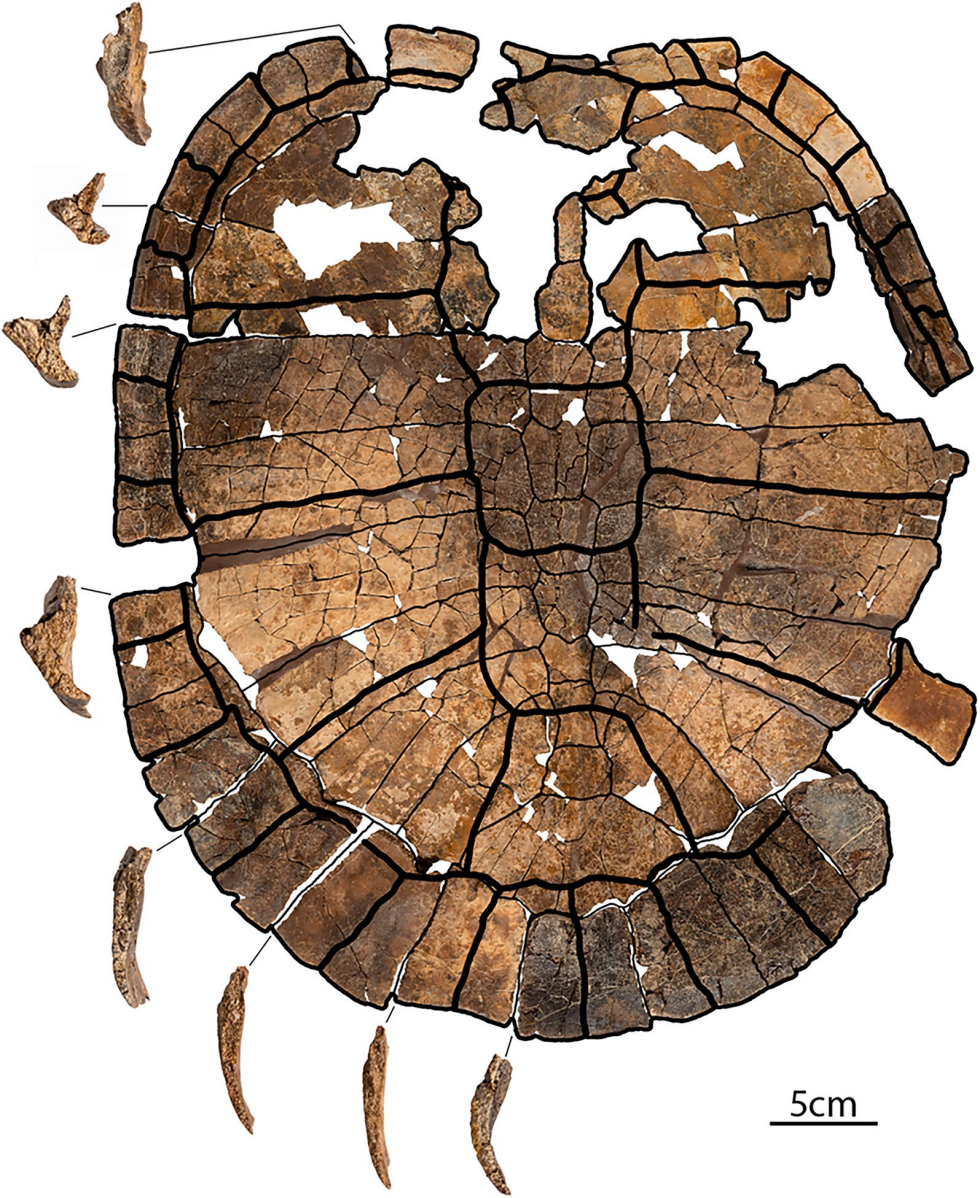
*Tavachelydra stevensoni*, gen. et sp. nov., DMNH EPV.141854 (DMNH Loc.19258), holotype, photograph overlain by line drawing of the sulci and photographs of the lateral edges of peripherals showing anteroposterior changes in cross sectional shape of peripherals. Lateral photos of peripherals were taken on the rostral edge and are oriented with their medial side up and the dorsal side to the right

**Fig. 5:**
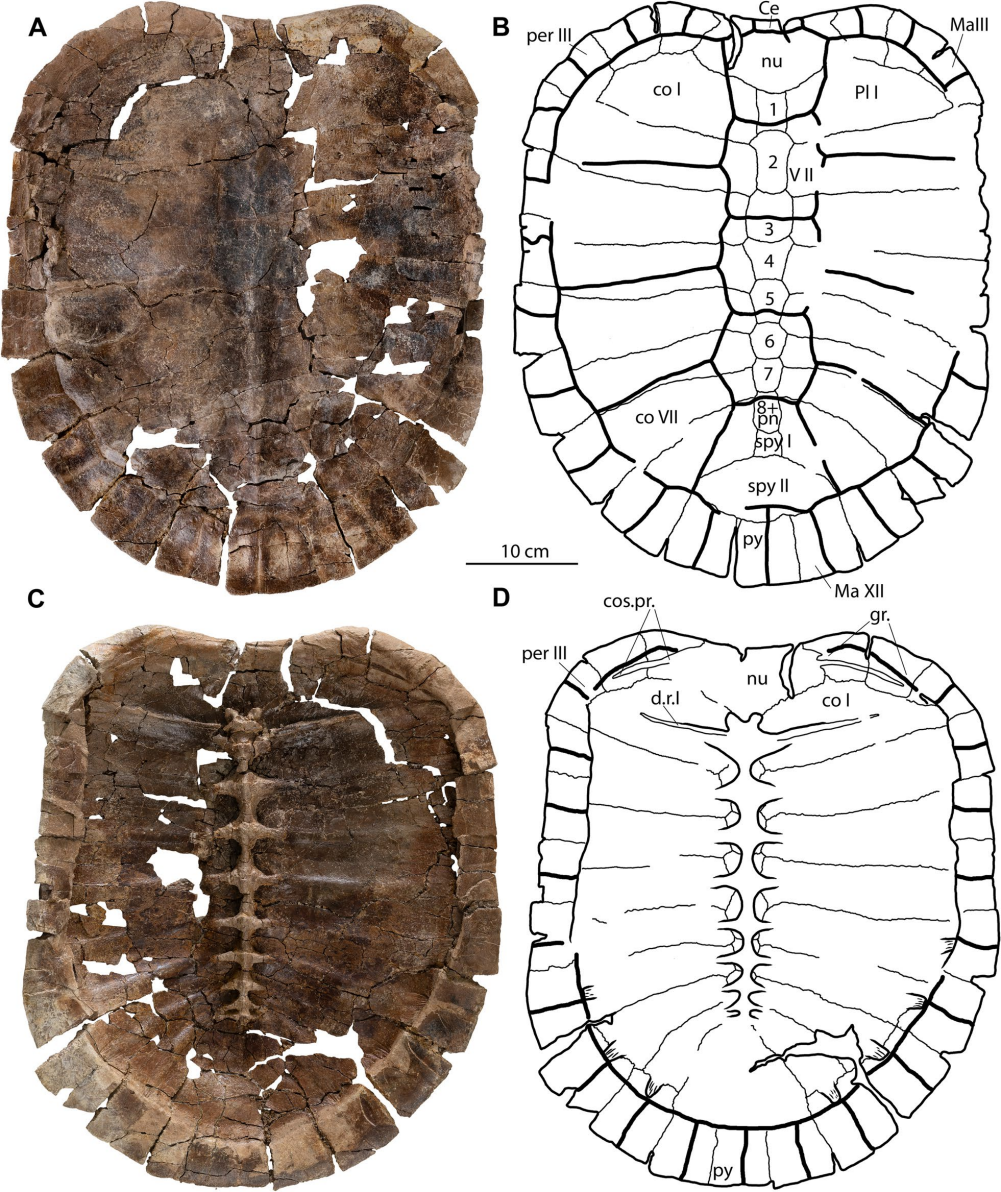
*Tavachelydra stevensoni*, gen. et sp. nov., DMNH EPV.143100 (DMNH Loc. 20,053), referred carapace. **A**, photograph and **B**, interpretive line drawing in external view. **C**, photograph and **D**, interpretive line drawing in internal view. *Ce* cervical scale, *co* costal, *cos.pr.* costiform process, *d.r.* dorsal rib, *gr.* groove, *Ma* marginal scale, *nu* nuchal, *per* peripheral, *Pl* pleural scale, *pn* postneural, *spy* suprapygal, *Ve* vertebral scale, *py* pygal. Arabic numerals denote neurals

**Fig. 6:**
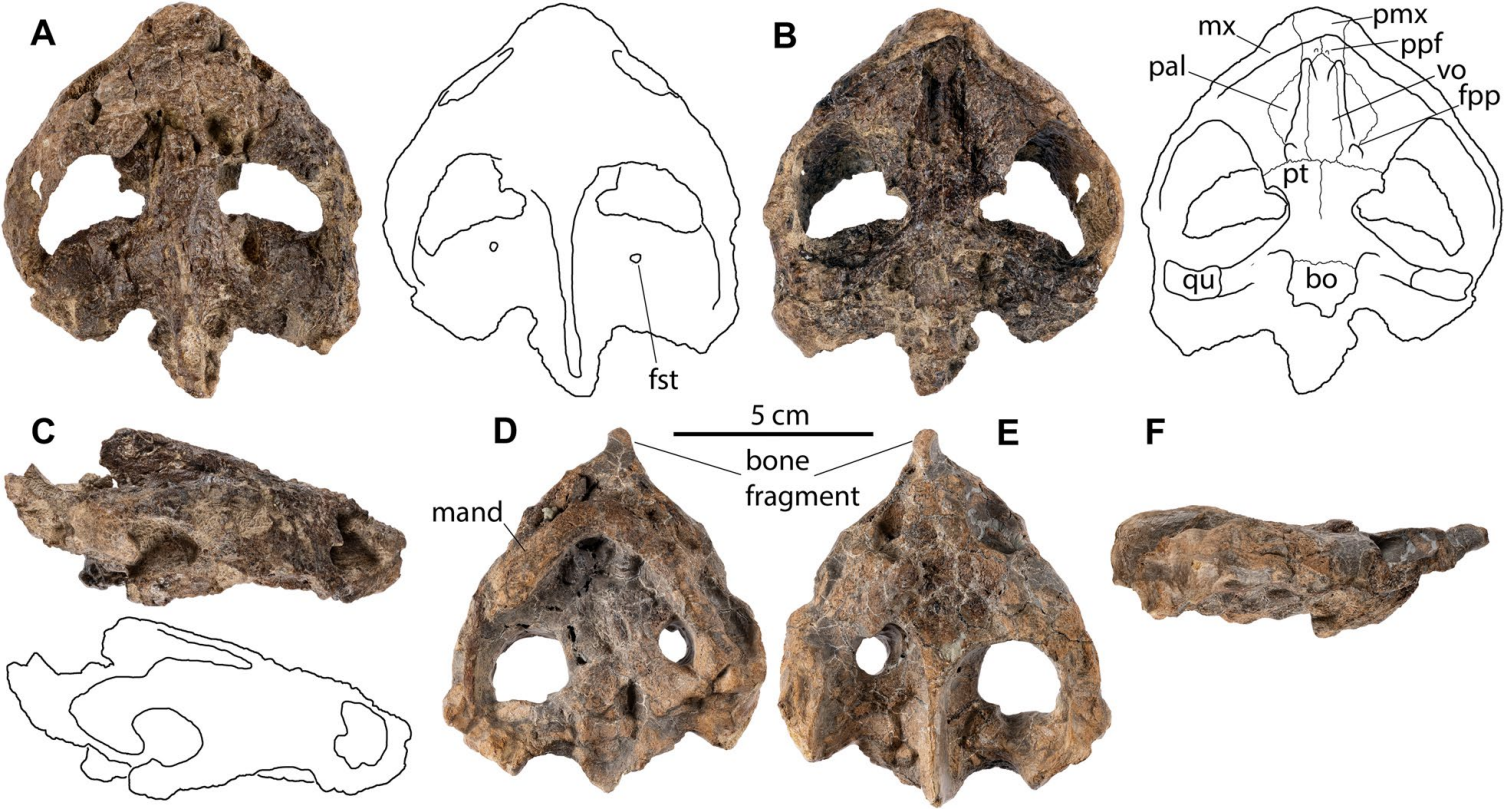
*Tavachelydra stevensoni*, gen. et sp. nov., referred crania. DMNH EPV.134087 (DMNH Loc.7082), **A**, photograph (left) and line drawing (right) of in dorsal view, **B**, photograph (left) and line drawing (right) in ventral view, **C**, photograph (top) and line drawing (bottom) in right lateral view. DMNH. EPV.136265 (DMNH Loc. 18,852), photographs in **D**, ventral, **E**, dorsal, and **F**, right lateral views. *bo* basioccipital, *fpp* foramen palatinum posterius, *fst* foramen stapedio-temporale, *mand* mandible, *mx* maxilla, *pal* palatine, *pmx* premaxilla, *ppf* praepalatine foramen, *pt* pterygoid, *qu* quadrate, *vo* vomer

**Fig. 7:**
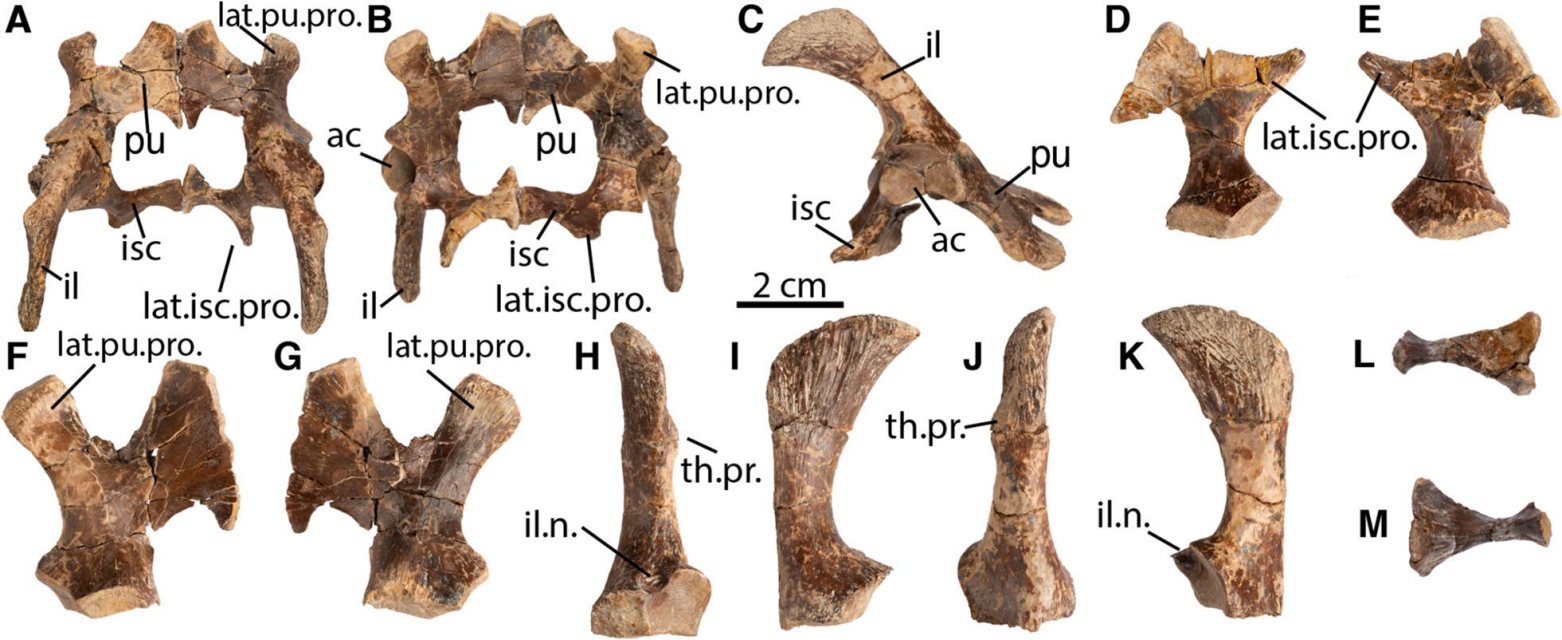
*Tavachelydra stevensoni*, gen. et sp. nov., DMNH EPV.141854 (DMNH Loc.19258), holotype, pelvis. Photographs of entire pelvis in **A**, dorsal, **B**, ventral, and **C**, right lateral views. Photographs of the right ischium in **D**, ventral and **E**, dorsal views. Photographs of the right pubis in **F**, ventral and **G**, dorsal views. Photographs of the right ilium in **H**, posterior, **I**, medial, **J**, anterior, and **K**, lateral views. Photograph of the right sacral rib in **L**, ventral and **M**, dorsal views. *ac* acetabulum, *il* ilium, *il.n*. ilial notch, *isch* ischium, *lat.isc.pro.* lateral ischial process, *lat.pu.pro.* lateral pubic process, *pu* pubis, *th.pr.* thelial process

***Tavachelydra stevensoni*** **, gen. et sp. nov.**

(Figs. 2, 3, 4, 5, 6, 7; also see 3D models (MorphoSource project ID: 000730318)).

**Type specimen:** DMNH EPV.141854, a disarticulated, but associated, skeleton that includes a nearly complete carapace and plastron and a complete pelvis (Fig. 2, 3, 4, 7). 3D model of the anterior region of the carapace is available at MorphoSource (project ID: 000730318).

**Type locality and age:** DMNH Loc. 19,258, Corral Bluffs Study Area, El Paso County, Colorado, USA. The Corral Bluffs Study Area is situated east of Colorado Springs (qualified researchers can obtain more detailed locality information from the vertebrate paleontology collections manager at the Denver Museum of Nature & Science; Fig. 1); Denver Formation, Danian, early Paleocene. The locality is 110.5 m above the pollen defined K/Pg boundary (Fuentes et al., 2019), within magnetochron 29n, and with a calculated age of 65.34my (GPTS2012 age model) or 65.37my (Clyde et al., 2016 age model). The holotype was collected from the top of the Puercan II NALMA within pollen biozone 2 (Fig. 1, point 1) and was found in a friable, silty mudstone unit rich in fossil plant fragments interpreted to be an overbank pond deposit that was frequently flooded.

**Etymology:** The name ‘Tavachelydra’ is derived from the combination ‘chelydra’ (Greek word ‘khéludros” which means “amphibious serpent”) and the morpheme ‘tava’ referring to one of the names that the Ute or Nuuchiu – a Native American people indigenous to the region of discovery – use for Pikes Peak (Tavá-Kaavi, direct translation “Sun Mountain” (Briggs, 2010)), which towers over the Corral Bluffs Study Area, having been combined following classical word formation for euphony. The species epithet honors the late Brandon D. Stevenson, a dear friend of T. R. Lyson and long-time supporter of the Corral Bluffs project.

**Nomenclatural acts:** This publication and its nomenclatural acts were registered at ZooBank on February 5, 2025, prior to publication. The LSID of the publication is urn:lsid:zoobank.org:pub:D622E60E-78EC-4F1F-A764-00DD98BE7F87, that of the new genus urn:lsid:zoobank.org:act:5AED1223-0AD4-4711-A6BB-7244E31708E2 and that of the new species urn:lsid:zoobank.org:act:37B5881D-5C1C-4B49-9E38-D6256C8620EE.

**Diagnosis:** *Tavachelydra stevensoni* can be diagnosed as a member of *Chelydroidea* (i.e., the clade consisting of *Kinosternoidea* and *Chelydridae*) based on the presence of a reduced cruciform plastron, strap-like epiplastra that cover the lateral margins of the hyoplastra, absence of extragular scales, absence of pectoral scales, absence of a midline contact between the abdominal scales, and less than four inframarginal scales; and as a representative of *Pan-Chelydridae* based on the presence of true rib-like costiform processes (sensu Joyce, 2007) that are not completely embedded in bone and that cross peripheral I and variably terminate in peripheral II or III on the visceral shell surface, the presence of a low carapace formed by thin neurals and costals, presence of intergular scales, strap-like xiphiplastra that cover the lateral margins of the hypoplastra, and an anal scale that is restricted to the posterior portions of the hypoplastra. *Tavachelydra stevensoni* differs from all other pan-chelydrid taxa in the following unique combination of characters: large size (straight carapace length of 45–50 cm), absence of plastral or carapacial fontanelles, smooth shell lacking radial plications, an upturned lip on the anterior margin of the nuchal, a thin and gracile plastron, an osseous bridge consisting of pegs and sockets but no sutures, a single, large intergular scale, a distinctive gutter in peripherals IV-VI, thin carapace lacking lateral keels on the costals, mid-line keel restricted to the posterior quarter of the carapace, presence of an iliac notch in the acetabulum, and a fan-shaped ilial shaft that is angled posteriorly and adorned by an incipient thelial process.

**Referred material and distribution: DMNH EPV.143100** (Fig. 5), an articulated complete carapace and partial right hyo- and hypoplastron found in situ 56 m from the holotype (DMNH EPV.141854). It originates from the same stratigraphic horizon (DMNH Loc. 20,053; Fig. 1, point 2) and is thus inferred to have the same age as the holotype specimen. A 3D model of the shell is available at MorphoSource (project ID: 000730318). **DMNH EPV.143200** (not figured), hypo- and xiphiplastra found in situ 97 m above the pollen calibrated K/Pg boundary (DMNH Loc. 6284; Fig. 1, point 5), within magnetochron 29n, and with a calculated age of 65.43 mya (GPTS, 2012 age model) or 65.49 mya (Clyde et al., 2016 age model). The specimen originates from the top of Puercan II NALMA and the middle of pollen zone P2 (Fig. 1, point 5). **DMNH. EPV.134087** (Fig. 6A, B, C), a poorly preserved, but complete cranium found as float and preserved in a fine grained phosphatic concretion. The specimen was found at the base of the bluffs, with approximately 45 m of section above (DMNH Loc. 7082; Fig. 1, point 3). Given that the specimen was fully intact, it likely did not travel far. The maximum age and stratigraphic position (i.e., the specimen was moved down section by gravity) was calculated for the specimen as 52 m above the pollen calibrated K/Pg boundary, within magnetochron 29r and with a calculated age of 65.74 mya (GPTS, 2012 age model) or 65.84 mya (Clyde et al., 2016 age model). This corresponds biostratigraphically to the top of the Puercan I NALMA and within the P1 pollen biozone (Fig. 1, point 3). **DMNH. EPV.136265** (Fig. 6D, E, F), a poorly preserved, but complete cranium and lower jaws found as float and preserved in a fine grained phosphatic concretion. The specimen was found on a knife ridge with approximately two meters of Paleocene section above (DMNH Loc. 18,852; Fig. 1, point 4). Given that the specimen was fully intact, the specimen likely did not travel far. In addition, there was only two meters of section above the specimen. The maximum age and stratigraphic position was calculated as 99 m above the pollen calibrated K/Pg boundary, within magnetochron 29n and with a calculated age of 65.41 mya (GPTS, 2012 age model) or 65.46 mya (Clyde et al., 2016 age model). Biostratigraphically the specimen is dated to the top of the Puercan II NALMA and within the P2 pollen biozone (Fig. 1, point 4).

## Description of material

**Shell**: The description of the shell is based primarily on the holotype (DMNH EPV.141854; Figs. Fig. 2, 3, 4) and the referred carapace (DMNH EPV.143100; Fig. 5). The anterior quarter of the holotype shell is preserved, but highly fragmented due to erosion, while the posterior three quarters of the shell is mostly complete. The complete left peripheral series is present, while the right peripheral series is missing peripherals XI and XII. The nuchal is preserved, but is damaged posteriorly. Costals I and II on both sides are preserved, but are highly fragmented, as are neurals I and II. The carapace was found partially articulated with the costals, neurals, and suprapygals found in articulation, but the elements on the perimeter (i.e., nuchal, peripherals, and pygal) were separated from the rest of the carapace. Several of the peripherals were articulated with one another. The hyoplastra, hypoplastra and xiphiplastra were all found in articulation, but the right epiplastra and entoplastron were separated from the rest of the plastron. The anteromedial portion of both hyoplastra is damaged due to erosion, as is the posterior portion of the entoplastron. The right epiplastron is complete and the left epiplastron is missing. A small portion between the plastron and articulated costals was preserved in a phosphatic concretion, but not the rest of the specimen. The carapace is less crushed and flattened where it was preserved in concretion and this portion suggests that the carapace was flat to slightly domed. A complete, disarticulated pelvis was found mixed in with the carapace and plastron elements. The referred carapace (DMNH EPV.143100; Fig. 5 and 3D models) was preserved intact and is more complete than the holotype carapace (DMNH EPV.141854). The left side of the referred carapace (DMNH EPV.143100) is largely complete, whereas the right side of the carapace is more fragmentary due to erosion. The right lateral portion of the hyo-hypoplastron was found as float alongside the carapace.

**Carapace**: The carapace is large, with a straight carapace length of approximately 45 cm for the holotype and 49.5 cm for the referred carapace (DMNH EPV.143100). The carapace is composed of a nuchal, eight neurals, two suprapygals, one pygal, eight pairs of costals and eleven pairs of peripherals (Figs. 2–5). A supernumerary postneural is present in the holotype and possibly in the referred specimen as well. The perimeter elements (nuchal, peripherals, and pygal) are distinctly thickened while the remaining carapace is thin, only 3–4 mm in thickness. The carapace is oblong in shape and lightly embayed anteriorly in the nuchal region. The sides of the shell are nearly straight and the posterior region is broadly rounded and lacks a caudal notch. The concreted portion, costals IV-VI, in the holotype specimen indicate that the shell is low domed. Narrow sulci are weakly impressed into the carapacial bones, but plications are absent. Peripherals IX, X, and XI are lightly scalloped posteriorly. Of the midline series, only the posterior midline neurals (VI – IX) and the suprapygal have a slight, continuous midline keel. Lateral keels are absent on the costals.

**Nuchal:** The nuchal is damaged posteriorly as well as on the left side in the holotype specimen, but is more complete in the referred carapace. The nuchal is a large trapezoidal structure, with an embayed anterior edge, two short margins that form a notched contact with peripheral I laterally, and longer margins that form a broadly-sloping contact with costal I, and a rounded contact with neural I posteriorly (Figs. 2A, B, 5A, B). The anterolateral margin of the nuchal is upturned to form a distinctive lip on either side of the midline cervical scale (see 3D models). The anterior edge of the nuchal is distinctively thickened but thins out posteriorly. Although both sides are damaged in the holotype, a long groove and remnants suggest that the costiform processes were rib-like and superficially crossed peripherals I and II and the anterior half of peripheral III, but the open nature of the groove precludes full confidence (Fig. 3C). In the referred carapace, by contrast, the costiform processes are more deeply imbedded into the peripherals and unambiguously terminate in the lateral portion of peripherals II (Fig. 5A, B, and 3D models). A broken portion of the left costiform process in the holotype shows that the process is oblong in cross section.

**Neurals:** Eight neurals are present in both specimens. A postneural is partially fused to neural VIII in the holotype and may be present in the referred specimen as well (Figs. 2A, B, 3A, B, 5A, B). A low, rounded median keel originates at neural III that becomes increasingly pronounced towards the posterior. Neural I is preserved completely in DMNH EPV.143100, has a rectangular shape, and contacts the nuchal anteriorly, costal I laterally, and neural II posteriorly (Fig. 5A, B). Neural II in the same specimen is an elongate subrectangular structure that forms a broadly-rounded contact with neural I and costal I anteriorly, contacts costal II laterally, and forms a straight contact with neural III posteriorly (Fig. 5A, B). In both specimens, neural III is hexagonal in shape with short anterior sides that contact costals II and long sides that contact costals III. The vertebral II/III sulcus is situated along the middle of neural III. Neural IV is hexagonal in shape with short anterior sides that contact costals III and long sides that contact costals IV. Neural IV is wider anteriorly and tapers slightly posteriorly where it forms a straight contact with neural V. Neural V is hexagonal in shape with short anterior sides that contact costals IV and long sides that contact costals V. Neural V is slightly shorter in length than neurals I–IV. The vertebral III/IV sulcus is situated on the posterior third of this neural. Neural VI is hexagonal in shape with short anterior sides that contact costals V and long sides that contact costals VI. Neural VI has a slight taper posteriorly where it forms a posteriorly-rounded contact with neural VII. Neural VII is hexagonal in shape and its width approximates its length. The short anterior sides contact costals VI and the long sides contact costals VII. A hexagonal neural VIII and a square postneural are fused in the holotype into an elongate, polygonal element. Although the two elements cannot be distinguished externally (Fig. 2A, B), a suture is apparent between the two in visceral view (Fig. 3A, B). The posterior of the two elements is identified as a postneural, as it is associated with dorsal vertebra X (see Menon and Joyce, in review, for discussion on homology). The neural VIII of the referred specimen has an irregular, elongate shape as well, which is highly suggestive of a fused element, but we cannot discern sutures in dorsal or ventral view. In either case, the fused neural VIII/postneural has short anterior sides that contact costal VII, elongate lateral sides that contact costal VIII laterally, and a short posterior contact with suprapygal I. The vertebral IV/V sulcus is situated on the middle (holotype) or anterior third of neural VIII (referred specimen).

**Suprapygal:** Two suprapygals (i.e. bones not associated with the vertebral column) are present in both available carapaces (Figs. 2A, B, 5A, B). Suprapygal I is nearly square, with its width just barely exceeding its length. Suprapygal I contacts costals VIII laterally, the postneural anteriorly, and suprapygal II posteriorly. Suprapygal II is a large crescent shaped element. On the right side of the holotype specimen, suprapygal II has a broad contact with peripheral XI and nearly contacts peripheral X, whereas on the left side it clearly only contacts peripheral XI. Corner contacts appear to be present on both sides of the referred specimen. The low, rounded midline keel that originates on the neurals becomes highly distinct in suprapygal I, fades along suprapygal II, and is absent on the pygal. The posterior margin of vertebral V is located on suprapygal II near its posterior suture with the pygal.

**Pygal:** The pygal is nearly square, with its length only slightly exceeding its width (Figs. 2, 5). The posterior half forms a distinctive trough or ridge, which is best seen in cross section (Fig. 4). The pygal is a thin element that tapers to a rounded point posteriorly.

**Costals:** Eight pairs of costals are present (Figs. 2, 3, 5). As in other turtles, the anterior five costals are larger than the posterior three costals. The costals are thin, no more than 3–4 mm thick. The intramembranous portion of the costals is tightly sutured with the peripherals leaving no space for carapacial fontanelles between the costals and peripherals. The endochondral rib portion of the costals inserts into peripherals III through X, best seen in the referred specimen (Fig. 5C, D). While most of the distal ends of the ribs are missing in the disarticulated holotype specimen, they are entirely preserved in left costals VI–VIII and about 1 cm in length, suggesting they insert deeply into each peripheral (Fig. 3A, B).

Costal I is the largest carapacial element, contacting peripherals I–III laterally, the nuchal anteromedially, neural I medially, and neural II posteromedially. The costal rib I terminates in peripheral III. The costal I/II suture contacts peripheral III. Ventrally, costal rib I (i.e., dorsal rib II) underlies the costiform process groove in peripheral III, suggesting that these two structures nearly touch in the holotype (Fig. 3C). In the referred carapace, the costiform process terminates at least on the surface in peripheral II and there is thus greater distance between it and the endochondral rib portion of costal I (Fig. 5C, D). Ventrally, the first dorsal rib, a thin and long strap-like structure that spans about half the width of costal I, is closely attached to the anteroventral portion of costal rib I (Figs. 3A, B, 5C, D). Costal II expands distally, contacts peripherals III and IV laterally, and its rib deeply inserts into the posterior half of peripheral IV. The costal II/III suture contacts peripheral V and the costal III/IV suture contacts peripheral VI. The width of costal III and IV does not change and their ribs insert into peripheral V and VI, respectively. Costal V expands distally and its rib inserts into the posterior half of peripheral VII. Costals VI–VIII are much smaller than the anterior costals and become successively smaller and shorter towards the posterior. Their ribs insert into the posterior halves of peripherals VIII, IX, and X respectively. The rib heads of dorsal ribs IX (i.e. costal rib VIII) and X are fully formed and medially contact the vertebral column. A pair of oblong depressions present on the ventral side of costal VIII are interpreted as the iliac scars, where the ilia articulate with the carapace (Fig. 3A, B).

**Peripherals:** Eleven pairs of peripherals are present (Figs. 2, 3, 4, 5). As in other pan-chelydrid turtles, the peripherals are robust, thick elements that form a broad rim around the otherwise thin carapace. Peripheral I is broadly triangular structure that is slightly longer than wide and contacts the nuchal medially, costal I posteriorly, and peripheral II laterally. The rib-like costiform process is loosely attached to the ventral side of peripheral I and II in a shallow groove in the holotype specimen (Fig. 3A, B, C) and is more deeply embedded in bone in the referred carapace (Figs. 5C, D), which we attribute to the larger and more ossified nature of the referred carapace. Ventrally the skin sulcus is visible midway along the length of peripheral II and the anterior portion of peripheral III. As in all turtles, costal ribs do not insert into peripherals I or II. The edge of the carapace is slightly upturned starting in peripheral II, becomes increasingly guttered through peripheral VI, but this flaring tapers to an end in peripheral VII (Fig. 4). The distal rib end of costal I inserts into the middle of peripheral III, cuts across this peripheral along a broad transverse groove (Fig. 3C), and ends at the posterior-most margin of this peripheral along the peripheral III/IV suture. Anteroventrally to this costal rib groove is a deep pit that receives the hyoplastral axillary buttress. The ventromedial edges of peripherals IV through VI have small, distinctive pits for articulation with the lateral pegs formed by the hyo- and hypoplastra (Fig. 3A, B; see 3D model for better view). The hypoplastral inguinal buttress ends in a pit in the middle of peripheral VII. The bridge peripherals are V- to C-shaped in cross section (Fig. 4). Peripherals VII through XI are long rectangular structures that are slightly upturned (Fig. 4). Peripherals III through X have distinct pits along their medial suture for articulation with the costal ribs. Such pits are not present on peripherals I, II, and XI. The posterior edges of peripherals IX through XI are weakly scalloped. Musk duct grooves are absent.

**Carapacial scales:** The carapacial sulci are overall only weakly impressed on the carapacial bones, but indicate the presence of five vertebrals, one undivided cervical, four pairs of pleurals, and twelve pairs of marginal scales. The cervical scale is small and approximately three times wider than it is long. It is entirely situated on the nuchal bone. Vertebral scale I is the smallest of the vertebral series. Its anterior width is slightly greater than its posterior width. The anterior two thirds of vertebral scale I is positioned on the nuchal, while the posterior third covers the anteromedial portion of costal I and the anterior two thirds of neural I. The vertebral I/II contact is located on the posterior portion of neural I. Vertebrals II, III, and IV are squarish in the holotype (Fig. 2), but slightly more hexagonal in the referred specimen (Fig. 5). The vertebral II/III and vertebral III/IV contacts are located on neural III and V, respectively. Vertebral V is trapezoidal in shape with its anterior width approximately half of its posterior width. The four pairs of pleural scales are large and rectangular to trapezoidal in shape. The pleural I/II, II/III, and III/IV sulci are situated on costals II, IV, and VI, respectively. The pleural IV and vertebral V sulcus transversely crosses costal VIII and the lateral-most corner of the suprapygal before contacting marginal XI. The marginal scales span the sutures between the peripheral series, starting with the nuchal/peripheral I suture through the peripheral XI/pygal suture. The marginal I–XI scales are restricted to the peripherals and do not extend onto the costals. The marginal XII scales, however, clearly extend anteriorly onto the suprapygal.

**Plastron:** The plastron in the holotype specimen (DMNH EPV.141854) consists of an entoplastron and paired epi-, hyo-, hypo-, and xiphiplastra (Fig. 2C, D, 3D, E). The plastron is mostly complete and is only missing the anterior portions of the hyoplastra and the complete left epiplastron. The plastron was found mostly articulated, with only the epiplastra and entoplastron disarticulated. It is approximately 30 cm long, significantly shorter than the straight midline length (45 cm long) of the associated carapace. The plastron lacks fontanelles and is thicker than the carapace, the lateral edges of the xiphiplastra having a thickness of 8–9 mm. The bridge is narrow, 35 mm in length at its narrowest point. The bridge has a loose osseous connection with the carapace via short pegs that insert from the middle of peripherals III to the middle of peripheral VII but does not extend onto the costals. Musk duct foramina are not present, but at least one deep, rounded depression along the lateral margin of the right hyoplastron is highly suggestive of a notch for passage of the musk duct. While the anterior plastral lobe is distinctly triangular, the posterior plastral lobe is more rounded.

**Epiplastra:** The epiplastron is a strap-like element that contacts its counterpart anteromedially, the entoplastron medially, and the hyoplastron posteriorly (Figs. 2C, D, 3D, E). The epiplastron forms a beak-shaped process along the anterior plastral lobe. The epiplastron medially forms broad grooves that embrace the rounded lateral margin of the entoplastron and hyoplastron. The full element forms a thick layer of metaplastic bone that fully obscures its origin as a clavicle.

**Entoplastron:** The entoplastron is an arrowhead-shaped element that is damaged posteriorly (Fig. 2C, D, 3D, E). It forms a thick layer of metaplastic bone that hides its origin as a T-shape interclavicle. Anteriorly the entoplastron forms a rounded tongue that fits into the groove formed by the epiplastron. It otherwise sutures with the hyoplastra via a poorly preserved peg-and-socket connection. The anterior plastron lobe thus lacks fontanelles.

**Hyo-/Hypoplastra:** The anterior edge of both hyoplastra is missing, but based on the epiplastra and entoplastron, it is clear that the hyoplastra anterolaterally form a tongue that sutures with the groove in the epiplastra and anteromedially forms distinct pegs that suture with the entoplastron (Fig. 2C, D, 3D, E). The hyoplastra form a straight suture with the hypoplastra across the middle of the plastron. The hyo- and hypoplastra suture with their counterparts along the plastral midline via a deeply interfingering suture. Posteriorly, the hypoplastron forms a broad posterolateral tongue that inserts into the xiphiplastron. This deeply interfingered contact is both visible in dorsal and ventral view (Fig. 2C, D, 3D, E). The minimum sagittal length along the hyo-/hypoplastra bridge is approximately 35 mm. Dorsally (i.e., internally), the lateral edges of the hyo-/hypoplastra are thickened to approximately 6–8 mm. Laterally, short pegs and ridges of the hyo- and hypoplastra connect with the peripherals. The axillary buttress inserts into the middle of peripheral III and the inguinal buttress is a long finger-like structure that inserts into the middle of peripheral VII. No portion of the hyo-/hypoplastra contacts the peripherals.

**Xiphiplastra:** The xiphiplastra are long, robust elements that form a sinuous contact with each other medially. The xiphiplastra are not tightly sutured to the hypoplastra anteromedially, but rather connect anterolaterally via a complex deeply interfingering connection. An elongate process of the xiphiplastron laterally covers the hypoplastron. Medial to this interfingering connection the xiphiplastra and hypoplastra are only loosely abutting another. Posteriorly the xiphiplastra create a distinctly pointed plastral lobe.

**Plastral scales:** We use the plastral nomenclature of Hutchison and Bramble (1981) as implemented by Joyce (2016) for pan-chelydrids. The plastral sulci are lightly impressed into the plastron and show the presence of an intergular scale, paired gular, humeral, abdominal, femoral, and anal scales, and three paired inframarginal scales. No anterior midline sulcus is present on the entoplastron, indicating that there is only one midline intergular. The intergular extends over the epiplastral beak, covering half of the epiplastra as well as nearly half of the entoplastron. The gular-humeral sulcus does not extend onto the epiplastra. The entirety of the humeral scale is therefore on the hyoplastron. Pectoral scales are absent. The abdominal scales do not contact another medially. The abdominal scales broadly contribute to both the axillary and inguinal notches. The humeral/femoral sulcus is confluent with the hyo/hypoplastron suture. The anal scales are paired and are restricted to the xiphiplastra. A postanal scale is absent. Three pairs of inframarginal scales are present. Inframarginal scales I and II are located on the hyoplastron and a larger third inframarginal scale on the hypoplastron. Inframarginal scale I is a small, triangular scale that covers the anterior tip of the axillary buttress. Inframarginal scale II is larger than I, is rectangular in shape, contacts inframarginal scale I and III anteriorly and posteriorly, respectively, the abdominal scale anteromedially, and participates in the axillary notch. Inframarginal scale III is a large, kite-shaped scale that contacts inframarginal scale II anteriorly, the abdominal scale anteromedially, and broadly participates in the inguinal notch posteromedially.

**Cranium:** The referred crania, DMNH EPV.134087 and 136,265, are complete, but are poorly preserved (Fig. 6). This type of preservation is common among vertebrate fossils from the Corral Bluffs Study Area preserved in phosphatic concretions (Lyson et al., 2019). Specimen DMNH EPV.134087 is the slightly larger of the two specimens and most of the description is derived from this specimen (Fig. 6A, B, C). This specimen is preserved three dimensionally, does not appear to be crushed, but the skull roof is particularly poorly preserved as the sutures on the dorsal skull roof are fully obliterated. In addition, the left squamosal, basioccipital region, and distal end of the supraoccipital are damaged. The smaller specimen, DMNH EPV.136265, is dorsoventrally compressed and has portions of the lower jaw preserved in occlusion with the cranium (Fig. 6D, E). An isolated bone fragment is preserved on the anterior tip of the skull giving the false impression that an elongate snout is present. This specimen is particularly poorly preserved as no sutures are visible.

The skull of DMNH EPV.134087 is large and triangular in shape with an occipital condyle to snout tip length of 96.9 mm and a maximum width of 99.8 mm. The supraoccipital extends posteriorly beyond the occipital condyle and, while damaged, the snout tip to distal supraoccipital length is 110 mm. The base of the supraoccipital flares out laterally to form a T-shaped cross section. The orbits are oriented vertically and the upper temporal emargination is deep extending well anterior of the otic chamber. The nasals slightly overhang the confluent nares. The orbit is large, more than three times larger than the confluent nares. A distinct notch is present in the posteroventral portion of the orbit on both sides of the skull, suggesting that this represents true morphology rather than taphonomic distortion. The cheek emargination is minor, not extending above the ventral-most portion of the orbit. Although damage makes them appear particularly large, the stapedial foramen is well-developed nonetheless. The triturating surface is broad and flat. The labial ridge is broadly rounded and formed by the maxillae and premaxillae. A lingual ridge of the triturating surface is absent. The palatines contribute broadly to the triturating surfaces. The *foramen praepalatinum* are situated entirely within the premaxillae. The vomer is a single, broad-bar-shaped element that contacts the premaxillae anteriorly, the maxillae anterolaterally, the palatines laterally, and the pterygoids posteriorly. The vomer floors the *apertura narium interna*.

**Pelvic girdle:** The complete pelvis is preserved in the holotype specimen (DMNH EPV.141854) and was found disarticulated and intermixed with the shell (Fig. 7). The pelvis broadly resembles that of other pan-chelydrids. The ilium, pubis, and ischium equally participate in the acetabulum. The acetabulum is a broad, bean-shaped structure. The ilium forms the dorsal third of the acetabulum, has a rod-shaped shaft, and expands dorsally into a fan-shaped structure. In lateral view, the ilium is posterodorsally directed and the expanded fan-shaped portion of the ilium has distinctive longitudinal ridges. The distal fan contacts the carapace along an elongate depression contained within costal VIII. A distinctive bump or thelial process is present midway up the rod-shaped shaft, but not associated with a kink in the ilial shaft. Like the distal fan, the thelial process has longitudinal ridges (Fig. 7). A distinctive iliac notch is present between the acetabulum and articular surface with the ischium, best seen in posterior view (Fig. 7H).

The ischium forms the posteroventral portion of the acetabulum. The medial plate of the ischium forms a broad plate that broadly contacts its counterpart medially. The lateral process of the ischium is large and distally exhibits longitudinal ridges. An anteromedial process of the ischium extends into the thyroid fenestrae. The merged thyroid fenestrae form a large opening shaped like a horizontal figure eight.

The pubis forms the anteroventral portion of the acetabulum. The lateral process of the pubis is elongate, oval in cross section, and has longitudinal ridges for most of its length. The pubes broadly meet along the midline to form a broad plate-like structure. A V-shaped and smooth groove between the pubes indicate the presence of an unossified epipubic process anteriorly. A small posterior process of the pubis extends into the thyroid fenestra, but does not contact the ischium.

The great lateral expansion of the pelvis suggests that the only preserved sacral rib can be identified as the first. It is unclear if additional sacral ribs articulate with the pelvis.

## Phylogenetic analysis and results

We modified the matrix of Joyce et al. (2022a), which in turn was modified from Joyce and Claude (2020), Lyson et al. (2017), and Knauss et al. (2011), to investigate the phylogenetic position of *Tavachelydra stevensoni*. The matrix was expanded through the addition of three characters: character 6, costiform process, if present: embedded in bone (0), superficially attached (1); character 37, bridge attachment: ligamentous only (0), osseous (1); and character 38, articulation if osseous bridge present: via pegs only (0), via pegs and sutures (1), sutures only (2). In addition, *Protochelydra zangerli* was rescored based on personal observations of numerous specimens from the Wannagan Creek Quarry, namely NDGS ND186 (hyo-/hypoplastra), SMM P75.22.271 (carapace and plastron), and SMM P76.28.259 (carapace and plastron). The updated matrix, which was compiled using Mesquite 3.81, consists of 29 taxa and 72 characters, includes all character state definitions, and is provided in Additional file 1. We analyzed the dataset in TNT v.1.6 (Goloboff & Morales, 2023). As in Joyce et al. (2022a), we ordered all characters that formed morphoclines (characters 5, 15, 23, 24, 38, 49, 51, 57, 64, 68, and 69, using regular counting). *Judithemys sukhanovi* Parham & Hutchison, 2003 was selected as the outgroup taxon for this analysis.

Contrary to Joyce et al. (2022a) and Joyce and Claude (2020) we did not exclude *Agomphus pectoralis* and *Cardichelyon rogerwoodi* from the analysis. While neither taxon is informative regarding pan-chelydrid relationships, our analysis recovered both species in stable phylogenetic positions. Initial runs of the analysis suggested *Tullochelys montana* to be ambiguously placed as either a pan-kinosternoid or a pan-chelydroid. Introduction of implied weighting (k = 12; Goloboff et al., 2018) added stability to the phylogenetic position of *T. montana* without interfering with placement *T. stevensoni* among pan-Chelydridae (Fig. S1).

**Fig. 8:**
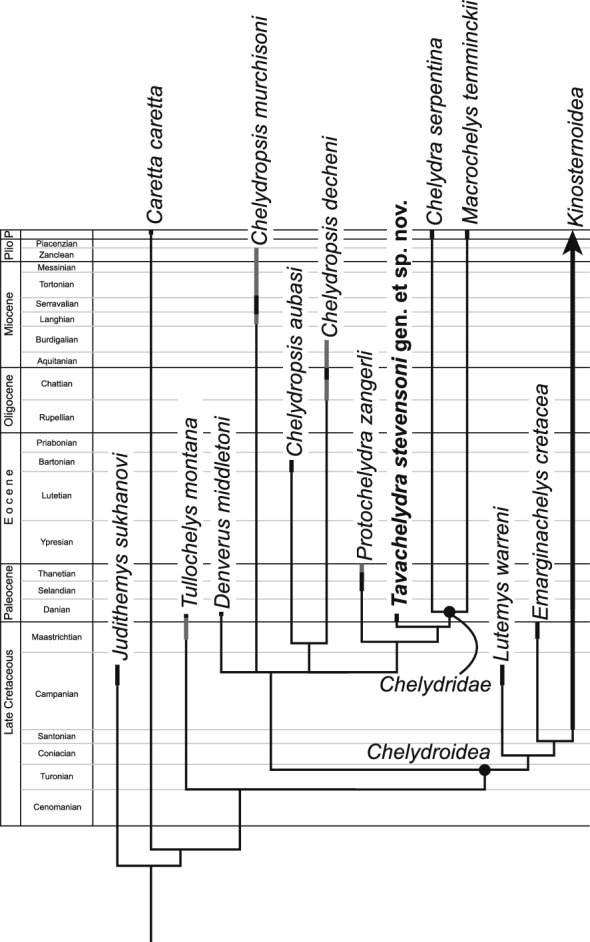
Cladogram of chelydroid turtles mapped against the stratigraphic ranges for each taxon (black = type strata, grey = age of referred material). Strict consensus tree from six most parsimonious trees (see Phylogenetic Methods)

The weighted dataset was analyzed using Traditional Search with 100,000 random addition sequences, followed by tree bisection and reconnection. We found that retention of 10 trees per iteration did not trigger replication overflow in TNT for this dataset, and manually increased tree space to hold a maximum of 1,000,000 trees. We report the implied weighting strict consensus tree (Fig. 8). See Additional file 2 for entire strict consensus tree using equal weighting (Fig. S1), strict consensus using implied weighting (Fig. S2), and synapomorphies mapped onto strict consensus tree (Fig. S3).

## Discussion

**Alpha taxonomy:** Several chelydroids comparable in age to *Tavachelydra stevensoni* have been described from the Great Plains and Rocky Mountain regions of North America, including *Emarginachelys cretacea* Whetstone, 1978, *Denverus middletoni* Hutchison & Holroyd, 2003, *Protochelydra zangerli* Erickson, 1973, and *Tullochelys montana* Hutchison, 2013. *Tavachelydra stevensoni* has several features, listed below, that distinguish it from each of these taxa and warrant recognition of a new pan-chelydrid turtle. For simplicity we focus on differences.

*Tavachelydra stevensoni* differs from *E. cretacea*, whose holotype is from the Hell Creek Formation (Lancian NALMA) of Montana (Whetstone, 1978), in the presence of a flat, smooth shell with broad peripherals, a low anterior gutter, and upturned nuchal lip (versus a highly domed shell with plications, narrow peripherals, no gutter, and no nuchal lip), presence of a posterior median carapacial keel, a bridge connected via pegs and sockets only (versus pegs and sockets and sutural connections), absence of a rib-like axillary process, termination of the inguinal buttress at the anterior half of peripheral VII (versus terminal on peripheral VIII), presence of narrow, strap-like epiplastra that form a triangular anterior plastral lobe (versus broad epiplastra that form a rounded anterior plastral lobe), absence of a post anal scale, formation of a macrocephalic skull with broad trituration surfaces that lack lingual ridges (versus an elongate skull with narrower trituration surfaces ornamented by distinct lingual ridges), and presence of a fan-shaped ilium that lacks a kink at midlevel of the shaft for the thelial process (Whetstone, 1978; pers. obs. of holotype).

*Tavachelydra stevensoni* differs from *P. zangerli*, whose holotype is from the Tiffanian NALMA of North Dakota (Erickson, 1973), in having an upturned nuchal lip, elongate costiform processes that are not deeply embedded in bone, a noticeably thinner plastron, presence of a thin strap-like epiplastron, attachment of the bridge via pegs and sockets only (versus pegs, sockets, and sutures), placement of the intergulars/gular sulcus on the entoplastron, restriction of the gular/humeral sulcus to the hyoplastron and anal scales to the xiphiplastra, and presence of a deep iliac notch (pers. obs. of all available material from the type locality held by SMM).

*Tavachelydra stevensoni* differs from *Tullochelys montana*, whose holotype is from the Puercan NALMA of Montana (Hutchison, 2013), in being significantly larger, having a longer costiform process that is not deeply embedded in bone, presence of a median keel that is restricted to the posterior third of the carapace, square shape of the vertebral scales, presence of a thin plastron, presence of a thin strap-like epiplastron, a larger metaplastic ossification on the entoplastron, attachment of the bridge via pegs and sockets only, placement of the intergulars/gular sulcus on the entoplastron, restriction of anal scales to xiphiplastra, absence of a post anal scale, and presence of three, instead of four, inframarginal scales.

*Denverus middletoni* is based on a partial shell that had also been collected from rocks exposed at Corral Bluffs dated to the Puercan NALMA (Hutchison & Holroyd, 2003). Since the description of *D. middletoni*, further biostratigraphic work has shown the holotype and only referred material is from the Puercan III NALMA (Lyson et al., 2019). Although comparisons are limited due to the highly fragmentary nature of the material, *T. stevensoni* differs from *D. middletoni* in being at least four times as large, presence of a smooth shell that lacks plications, and two inframarginals that are contained on the hyoplastron (Hutchison & Holroyd, 2003, pers. obs. of holotype). Given that the shell of extant juvenile chelydrids is smoother than that of the adult and that most turtles have a tendency to reduce the relative width and hexagonal shape of their vertebrals (Joyce et al., 2022b), differences in shell sculpturing and vertebral widths could be attributed to ontogeny, with the remaining differences representing intraspecific variation. We note, nonetheless, that *T. stevensoni* differs significantly from *D. middletoni* by exhibiting a proportionally much narrower bridge. Indeed, the bridge of *T. stevensoni* is about equal in width to the bridge of *D. middletoni*, even though the plastron is at least four times as large. As attribution to the same species would imply extremely allometric growth for this region of the shell alone and as other, coeval chelydroids are known to have wide bridges as well, we find difference in bridge width sufficient to support referral of the new material to a separate taxon.

**Phylogenetic Relationships:** Our phylogenetic analysis recovered 6 most parsimonious trees with a tree score of 8.0331 (217 character-state changes, consistency index = 0.453, retention index = 0.705; see Fig. 8 for strict consensus tree). The strict consensus tree is broadly consistent with that of previous phylogenetic analyses using prior versions of the matrix used herein (Joyce & Claude, 2020; Joyce et al., 2022a; Knauss et al., 2011; Lyson et al., 2017). Similar to previous analyses, *Emarginachelys cretacea* is regarded as the basal-most stem kinosternoid. A notable difference is that our analysis, unlike previous analyses, resolves the placement of *Tullochelys montana* as a stem chelydroid. Finally, while not fully resolved, our analysis provides more resolution within pan-Chelydridae than Joyce et al. (2022a) in that *Chelydropsis* is not monophyletic and in that *Chelydropsis murchisoni* and *Denverus middletoni* form a polytomy with the remaining pan-chelydrids.

*Tavachelydra stevensoni* is recovered as the immediate sister to crown *Chelydridae*, which is only composed of the extant *Chelydra serpentina* and *Macrochelys temminckii*. This arrangement is supported by a superficially attached costiform process (Ch. 6) and a strap-like or thin epiplastron (Ch. 33), the latter is also found in *Lutemys warreni* and *Judithemys sukhanovi*. The slightly younger *Protochelydra zangerli* is sister to the *T. stevensoni*/crown chelydrid clade, a grouping supported by more or less square vertebrals II-IV (Ch. 27). Finally, the European *Chelydropsis decheni* and *Chelydropsis aubasi* help form the next more inclusive clade, supported by presence of a median keel that consists of multiple posteriorly-fading subkeels originating at the intervertebral sulci (Ch. 8) and three inframarginals (Ch. 57). They share the former character with the kinosternoid *Hoplochelys clark* and the latter character with the crown-chelydrid *C. serpentina* and the clade Kinosternidae. Pan-Chelydridae is united by presence of a median keel that is developed only along the posterior part of the shell (Ch. 7), presence of a beak-like process along the anterior plastral lobe (Ch. 31), a hypoplastral buttress that extends posteriorly maximally to peripheral VII (Ch. 42), and the absence of lingual ridges (Ch. 61). Chelydroidea is united by a large thickened metaplastic bone of the entoplastron (Ch. 36).

**Ecology:** *Tavachelydra stevensoni* is a relatively rare component of the early Paleocene Puercan aquatic assemblage of the Denver Basin. Aside from the two referred shells and two referred crania, no other material has been found at any of the microsites at Corral Bluffs, or other prolific early Paleocene localities (i.e., Alexander Locality, South Table Mountain, West Bijou) within the Denver Basin. *Hoplochelys clark* Knauss et al., 2011, *Axestemys infernalis* Joyce et al., 2019, *Adocus* spp., *Compsemys victa*, and baenid turtles are all much more common in the Denver Basin, both as complete shells and crania, as well as isolated elements in microsites. The rarity could be a taphonomic artifact as much of the shell of *T. stevensoni* is thin, although the bones along the perimeter of the shell (nuchal, peripherals, and pygal) are notably robust. Interestingly, *Hoplochelys clark* has a similarly thin shell with robust peripheral elements and its peripherals are a common component of the Corral Bluffs microsites. Thus, the rarity of *T. stevensoni* is likely not taphonomic and reflects a true ecological signal.

**Fig. 9:**
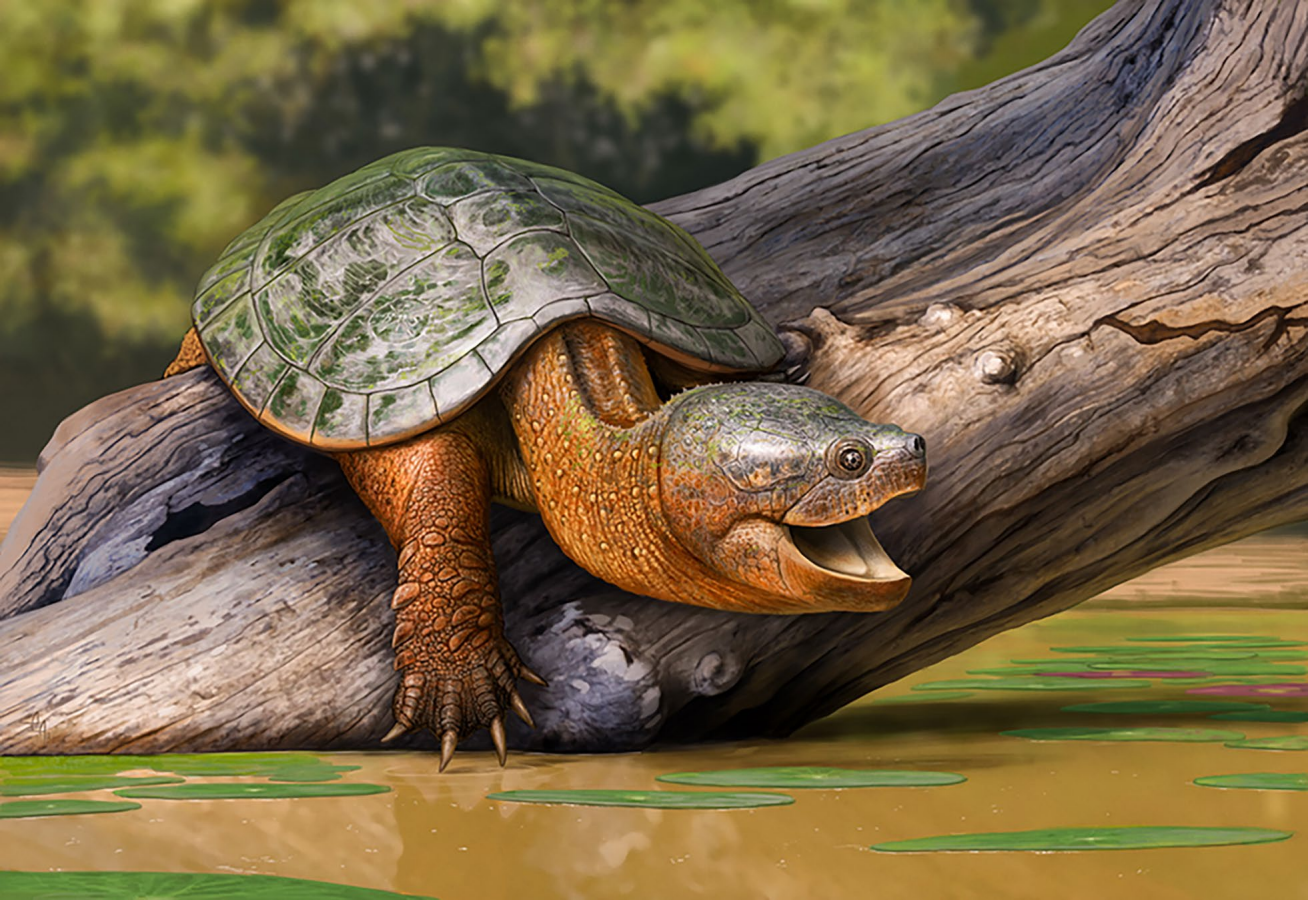
A reconstruction of *Tavachelydra stevensoni* gen. et sp. nov. basking on a log in a ponded water environment. Artwork by Andrey Atuchin

With a straight carapace length of nearly 50 cm, *T. stevensoni* is one of the larger turtles within the early Paleocene (Puercan) of the Denver Basin, with only *A. infernalis* being larger and *Neurankylus* sp. being comparable in size. Interestingly, two pan-chelydrid turtles are present in the early Paleocene of Corral Bluffs, *Tavachelydra stevensoni* and *Denverus middletoni*. The two differ significantly in size, with *T. stevensoni* being at least four times larger than *D. middletoni*, which likely helped reduce competition between two chelydrids with overlapping geographic ranges. Little is known about the paleoenvironment of the single known specimen of *D. middletoni*, but the fine- to medium-grained mustard yellow sandstone in which it was found was initially interpreted to represent a channel infill (Hutchison & Holroyd, 2003). The two partial *T. stevensoni* skeletons described herein, by contrast, were found near one another in a ponded water or distal crevasse splay deposit, so it is reasonable to infer that *T. stevensoni* inhabited ponded water overbank deposits (Fig. 9). While nothing can be said about the diet of *D. middletoni* as its skull is not preserved, *T. stevensoni* had a large skull with broad, flat triturating surfaces indicative of a durophageous diet. It therefore appears plausible that *D. middletoni* and *T. stevensoni* occupied different ecological niches, with *T. stevensoni* preferring ponded environments and a durophagous diet (Fig. 9).

Interestingly, turtle taxa with a durophageous diet have a higher survivorship across the K/Pg mass extinction compared to turtles with a non-durophageous diet (Lyson & Joyce, 2009). In addition to the inferred durophagous dietary preferences of *T. stevensoni*, several of the more common reptiles described from Corral Bluffs are also interpreted to be durophagous, including the button-toothed crocodylian cf. *Wannaganosuchus*, and the baenid turtles *Palatobaena knellerorum*, *Saxochelys gilberti*, and *Cedrobaena putorius* (Lyson et al., 2019). This suggests durophagy was an important life-history trait for freshwater vertebrates during the earliest Paleocene of North America.

## Supplementary Information

Additional file 1. Character-taxon matrix.

Additional file 2. Supplementary figures

## Data Availability

All 3D models are available at MorphoSource (https://www.morphosource.org/projects/000730318).

## Abbreviations

DMNH: Denver Museum of Nature & Science, Denver, Colorado, USA
NDGS: North Dakota Geological Survey, Bismarck, North Dakota, USA
SMM: Science Museum of Minnesota, Saint Paul, Minnesota, USA
