# Supplementary material for: A new pan-chelydrid turtle, Tavachelydra stevensoni gen. et sp. nov., from the lower Paleocene (early Danian, Puercan) Corral Bluffs Study Area in the Denver Basin, Colorado
Source: Swiss J Palaeontol. 2025 Aug 5;144(1):46. doi: 10.1186/s13358-025-00375-4 (PMC12325531; doi:10.1186/s13358-025-00375-4)
Supplement: Supplementary file 2 — Additional file 2. Supplementary figures [file 13358_2025_375_MOESM2_ESM.docx]

Journal of Vertebrate Paleontology

A new pan-chelydrid turtle, *Tavachelydra stevensoni* sp. nov., from the lower Paleocene (early Danian) Corral Bluffs Study Area in the Denver Basin, Colorado

TYLER R. LYSON, SALVADOR BASTIEN, HOLGER PETERMANN, NATALIE TOTH, EVAN TAMEZ-GALVAN, SADIE SHERMAN, and WALTER G. JOYCE


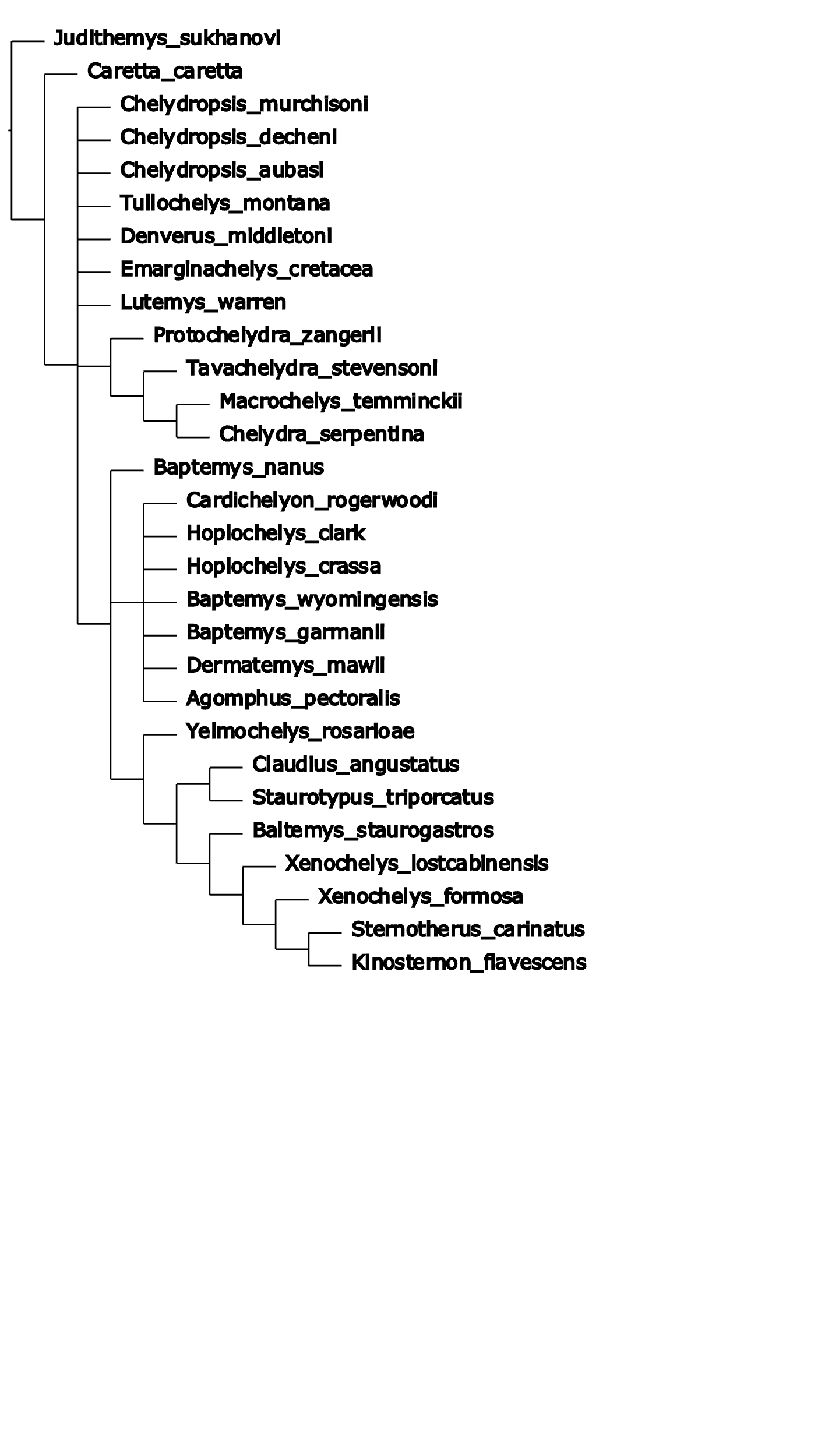


Figure S1: Strict consensus tree of the unweighted maximum parsimony analysis. The analysis was performed with 100,000 random addition sequences and TBR, keeping up to 100 trees per repetition, and resulting in 82 most parsimonious trees with a tree length of 214.


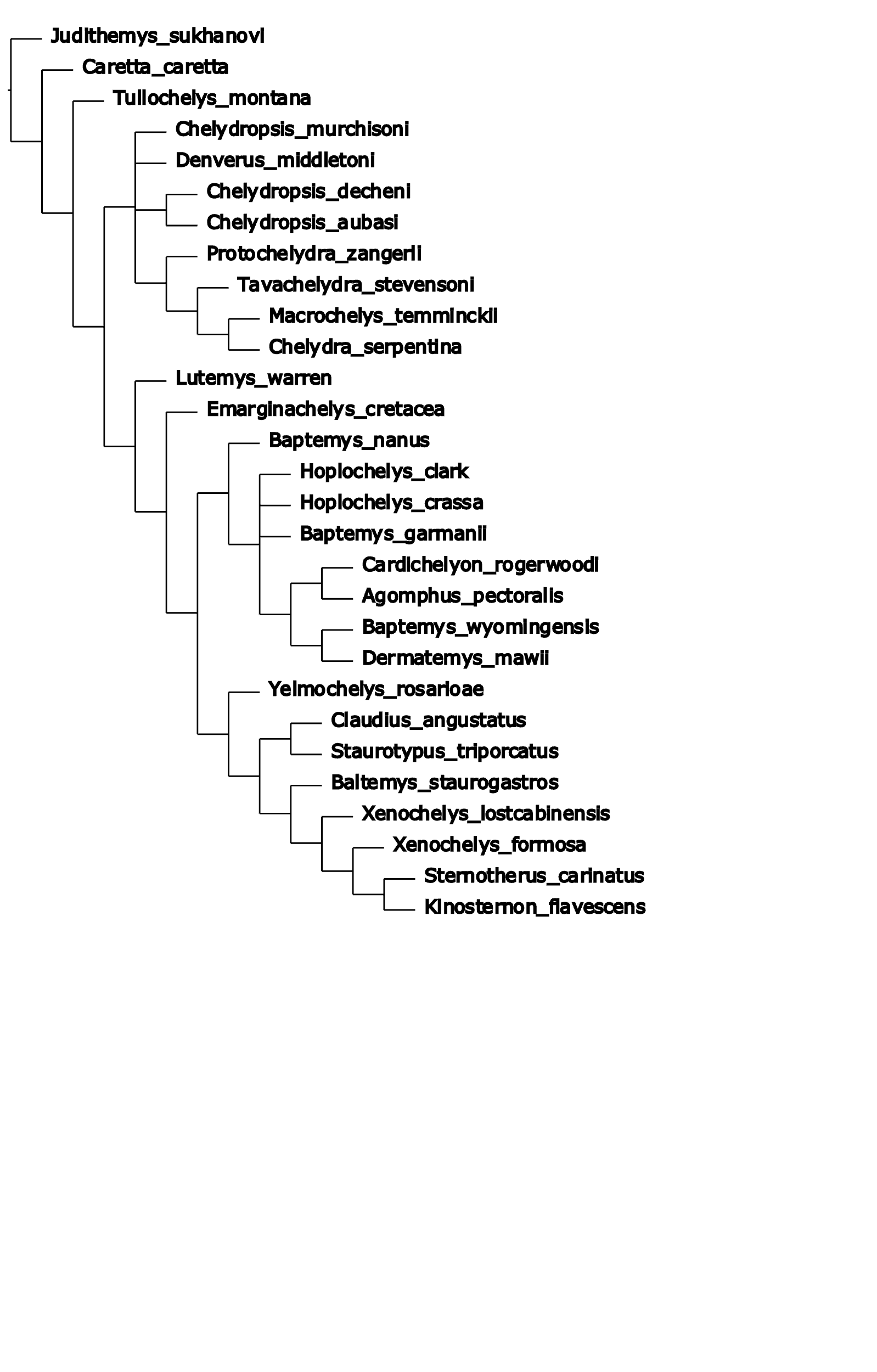


Figure S2: Strict consensus (implied weighting analysis) tree of six most parsimonious trees.


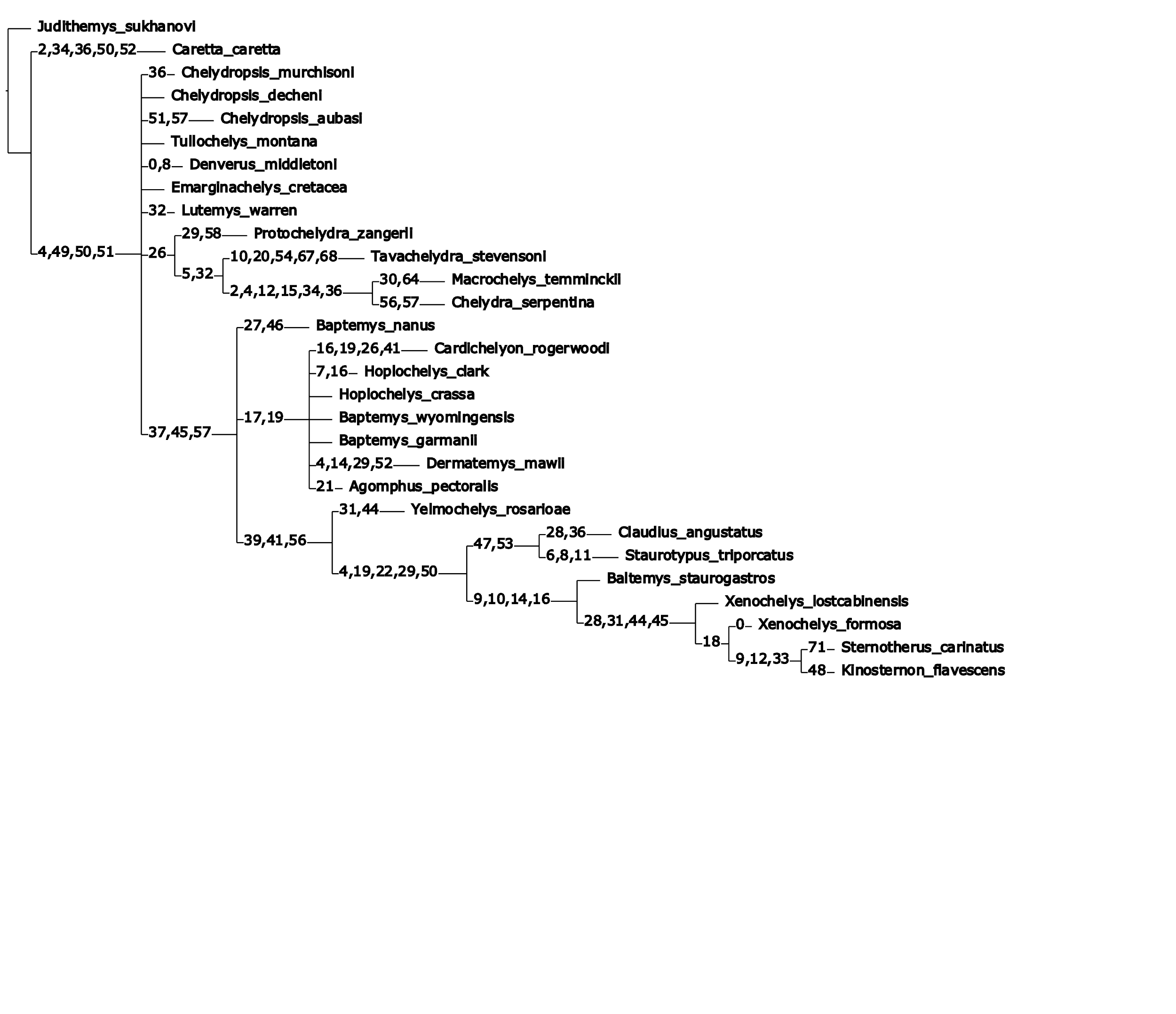
Figure S3: Synapomorphies mapped onto the strict consensus tree. Numbers at each node refer to phylogenetic characters that can be found in the morphologic data matrix.
